# Head Acceleration Event Probabilities During Standardised Contact Drills in Academy Men's Rugby League

**DOI:** 10.1002/ejsc.70232

**Published:** 2026-08-08

**Authors:** Ryan White, Daniel Weaving, Cameron Owen, Greg Roe, Thomas Sawczuk, James Tooby, Gemma Phillips, Dane Vishnubala, Paul Anderson, Ben Jones

**Affiliations:** ^1^ Carnegie Applied Rugby Research (CARR) Centre Carnegie School of Sport Leeds Beckett University Leeds UK; ^2^ Sport Science and Medicine Department Crystal Palace FC London UK; ^3^ Department of Physical Activity and Sport Faculty of Arts and Sciences Edge Hill University Ormskirk UK; ^4^ Applied Sports Science and Exercise Testing Laboratory The University of Newcastle Ourimbah New South Wales Australia; ^5^ Rugby Football League Manchester UK; ^6^ Uni‐X Mobility Cycling Oslo Norway; ^7^ Faculty of Biological Sciences School of Biomedical Sciences University of Leeds Leeds UK; ^8^ PREM Rugby London UK; ^9^ School of Behavioural and Health Sciences Faculty of Health Sciences Australian Catholic University Brisbane Queensland Australia; ^10^ Division of Exercise Science and Sports Medicine Department of Human Biology Faculty of Health Sciences the University of Cape Town and the Sports Science Institute of South Africa Cape Town South Africa

**Keywords:** head‐acceleration‐event, instrumented mouthguards, microtechnology

## Abstract

This study investigated head acceleration event (HAE) probabilities between common contact‐drills in rugby league. Fifteen professional male academy players participated in three training sessions, completing standardised contact‐drills while wearing custom‐fitted iMGs, all sessions were filmed. HAE data (peak linear acceleration and peak angular acceleration) were captured, synchronised, and processed alongside video data. Binomial logistic regression and ordinal mixed‐effect regression models were used to calculate overall and exceedance probabilities. Data included 1402 (93 ± 50 per player) contact‐drill observations, of which 133 had HAEs ≥ 5 *g* (9 ± 8 per player). Substantial variability in probability for HAEs ≥ 5 *g* (1.3%–41.3%) was observed across drills. Standing wrestle had the highest HAE ≥ 5 *g* probability (41.3%; CI: 31.0%–52.3%), while tackle shield hits at 1 m had the lowest (1.3%; CI: 0.5%–3.4%). HAE probability was greater for tacklers than ball‐carriers. Overall, probability of a HAE ≥ 25 *g* was < 1.1%. Standardised contact‐drills varied in HAE probabilities, but chances of higher magnitude HAEs was low for all drills. Overall, the probability of a HAE during standardised contact‐drills was low, although practitioners should consider the differences (i.e., the variability) in HAE probabilities when managing HAE training exposure.

## Highlights

- HAE ≥ 5 *g* probability during standardised rugby league contact drills was low, with only 9% of drill observations resulting in a HAE, indicating most contact exposures are unlikely to produce measurable head accelerations.
- HAE probability varied substantially across drills (1.3%–41.3%), with standing wrestle showing the highest likelihood, suggesting prolonged grappling and repeated contacts disproportionately contribute to head acceleration exposure.
- Higher‐magnitude HAEs (≥ 25 *g*) were rare (< 1.1%) across all drills; however, tacklers consistently demonstrated greater HAE probability than ball‐carriers, highlighting role‐specific exposure differences within standardised training formats.
- Profiling drill‐specific HAE exposure may provide a more effective framework for managing contact load distribution than attempting to reduce exposure through isolated drill modification alone.

## 1 Introduction

Rugby league involves frequent collisions between players (Naughton et al. 2020), with relatively high concussion rates reported in matches (15.5/1000 hours) (Eastwood et al. 2023). The tackle event is key for match success which provides the primary contact element in the sport (Naughton et al. 2020). Therefore, players are exposed to contact training (e.g., tackling and wrestling) to prepare for matches (Parmley et al. 2024). Head acceleration events (HAEs), defined as acceleration responses of the head caused by short‐duration external collision forces (Nguyen et al. 2019; Tierney et al. 2020; Kuo et al. 2022), can occur during the tackle event (Roe, Sawczuk, Owen, et al. 2024). Globally, sports are proactively aiming to reduce concussions and HAEs (Hendricks et al. 2023), given the potential dose‐response association with neurodegenerative diseases (Daneshvar et al. 2023; Patricios et al. 2023; Stewart et al. 2023). Thus, it is essential for athletes' welfare that HAE exposure is investigated further.

Instrumented mouthguards (iMGs) are a novel technology that provide valid measures of head kinematics and enable the quantification of HAEs arising from both direct head impacts and inertial body loading in sport (Jones et al. 2022; Tooby, Till, et al. 2024). Recently, the Rugby Football League has introduced contact‐load guidance to directly influence the volume and intensity of contact exposure during senior‐level training (i.e., the Men's and Women's Super League) (Parmley et al. 2024). Quantifying HAEs within training drills and understanding how they accumulate across sessions is critical, particularly given the hypothesised dose–response relationship between cumulative head impact exposure and long‐term neurological risk (Daneshvar et al. 2023). Data relating to HAE exposure during matches is emerging in rugby league (Tooby et al. 2025). Mean HAE values from match‐play, in the form of position‐specific incidence rates (i.e., mean count of HAEs per standardised playing time) and probability of exceeding specific magnitude thresholds, may offer useful benchmark reference points for contextualising an individual player's relative exposure during training (Tooby et al. 2025). Recent evidence suggests that, although overall HAE incidence rates during training are substantially lower than match‐play, a small proportion of relatively high‐magnitude HAEs still occur during training and may represent key targets for preventative intervention (Parmley et al. 2025). However, there remains a lack of detailed data on HAEs during training, limiting practitioners' ability to monitor, manage, and periodise head acceleration exposure in applied settings, particularly within academy rugby league, where the existing literature is sparse. This is important, as the training environment is arguably more modifiable than matches, given the greater proportion of time spent training relative to match play.

A particular challenge during contact training is that practitioners manipulate different constraints (e.g., number of players, area of pitch per player, protective equipment, and contact intensity), which are likely to influence the probability of players experiencing HAEs. Therefore, it is important to understand potential differences in HAE probability between common contact training drills and their associated constraints. Identifying drills associated with higher HAE burdens, alongside lower‐risk alternatives, would provide benchmark data to support practitioners in planning and periodising contact training. Such information may allow coaches and policy makers to substitute higher‐risk activities with drills that achieve similar performance or technical objectives while mitigating unnecessary HAE exposure. Consequently, this study aimed to describe the HAEs observed during standardised contact training drills used in professional academy rugby league.

## 2 Methods

### 2.1 Recruitment

All 12 Super League academies were approached by the Rugby Football League (RFL), the sport's governing body, and provided with a detailed project outline. This included the study's aims, an overview of the methods, and a summary of the potential benefits and limitations of participation. An information session was scheduled for clubs that expressed interest, allowing for further discussion and clarification. Of the 12 Super League clubs contacted and consulted, only one club was able to formally commit to participating in the study due to a mix of logistical constraints, scheduling commitments, or lack of interest. As a result, the sample for this study was one of convenience, reflecting the practical realities and challenges of conducting research in applied elite sport environments.

### 2.2 Participants

Fifteen male academy rugby league players (age: 17 yrs ± 4 months; height 182 ± 9 cm; body mass 84 ± 10 kg) from the same professional Super League club participated. Players completed three in‐season training sessions that formed part of the club's normal, coach‐led training programme and were not manipulated specifically for the purposes of this study. The standardised contact drills were completed post‐warm‐up, in the same order in each session, and were delivered as they would be within a typical applied training environment. Players completed seven standardised drills in the same order in each session. This fixed order was chosen to ensure a progressive increase in contact intensity and drill complexity, moving from structured to more chaotic scenarios, in line with typical coaching practice and applied training design. Players wore a custom‐fitted iMG (V1.4, Prevent Biometrics—Minneapolis, MN, USA), and each session was filmed. Ethics approval was received from the University Ethics Committee, with players providing informed consent (REF #108638).

### 2.3 Instrumented Mouthguards

All participants underwent three‐dimensional dental scans and were provided with custom‐fit iMGs (Prevent Biometrics, Minneapolis, MN, USA; Version 1.4). Each iMG housed triaxial linear accelerometers and gyroscopes sampling at 3200 Hz, with measurement ranges of ± 200 *g* and ± 35 rad·s^−1^, respectively (Jones et al. 2022). Coupling of the iMG to the upper dentition was monitored using embedded infrared proximity sensors, which confirmed periods when the mouthguard was worn (‘on‐teeth’ status) and enabled continuous monitoring of head kinematics (Tooby, Woodward, Tucker, et al. 2024). Proximity sensor data were processed in‐house by Prevent Biometrics and provided time‐stamped records (HH:MM:SS) of valid wear periods, allowing alignment with synchronised video footage for subsequent verification procedures. In addition, each detected HAE was recorded with a real‐world timestamp (HH:MM:SS), enabling direct temporal alignment between HAE occurrence and video footage. Laboratory validation of the Prevent Biometrics iMG has demonstrated excellent agreement with reference sensors (concordance correlation coefficient = 0.98; 95% CI: 0.98–0.99), while field‐based video‐verification studies in rugby league have reported a positive predictive value of 0.94 (0.92–0.95) and sensitivity of 0.75 (0.67–0.83) (Jones et al. 2022).

The iMGs were configured with an event trigger threshold of 8 *g* along any accelerometer axis, which initiated the recording of a HAE (Parmley et al. 2025). For each HAE, a discretised kinematic window comprising 10 ms of pre‐trigger and 40 ms of post‐trigger data was recorded, resulting in a 50 ms waveform centred on the trigger point. Linear acceleration data were transformed to the estimated head centre of gravity (CoG) using the relative acceleration equation, assuming a rigid‐body model of the head. The CoG location was defined using a 50th‐percentile male head model, consistent with previous iMG validation and application studies (Kuo et al. 2016). Angular kinematics were not transformed, in line with established methodological approaches (Roe, Sawczuk, Tooby, et al. 2024). Linear acceleration was directly measured by the accelerometer, while angular acceleration was derived via numerical differentiation of the angular velocity signal recorded by the gyroscope (Roe, Sawczuk, Tooby, et al. 2024).

All linear and angular kinematic data were initially processed by Prevent Biometrics using a four‐pole, zero‐phase, low‐pass Butterworth filter with a 200 Hz cut‐off frequency. HAEs were subsequently classified as true or false positives using Prevent Biometrics' in‐house event discrimination algorithm, based on infrared proximity sensor data (confirmation of on‐teeth coupling) and kinematic signal characteristics, including waveform shape and noise content (Jones et al. 2022). Each HAE was also assigned a signal‐quality classification by the Prevent Biometrics algorithm, categorising events as minimal (*n* = 116), moderate (*n* = 14), or severe (*n* = 3) noise. An additional filter was applied to HAEs with moderate and severe noise with cut‐off frequencies of 100 and 50 Hz, respectively. All HAEs, irrespective of noise classification, were retained for analysis following this processing. Peak linear acceleration (PLA; g) and peak angular acceleration (PAA; rad·s^−2^) were calculated as the maximum resultant values extracted from the 50 ms waveform for each HAE, representing event magnitude. Finally, only HAEs exceeding both 5 *g* and 400 rad·s^−2^ were included in the final dataset to minimise the inclusion of non‐contact events, in line with previous recommendations (Tooby et al. 2022; Tooby et al. 2024).

### 2.4 Standardised Drill Prescription

**FIGURE 1:**
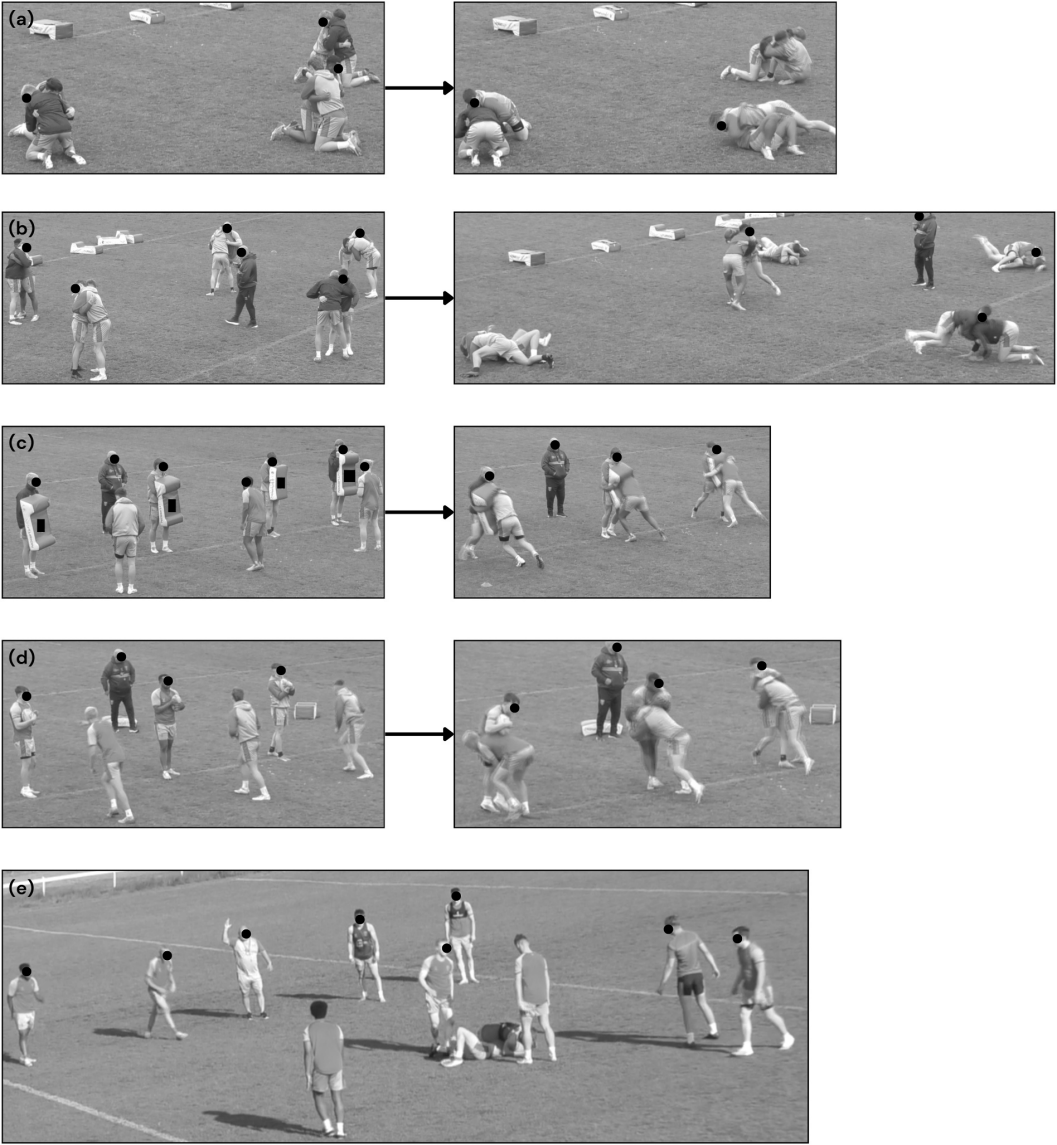
Visual examples of the standardised training drills performed in order. (a) Kneeling wrestle, (b) standing wrestle, (c) tackle shield hits, (d) ball‐carrier hits, and (e) ruck drill. Tackle shield hits and ball‐carrier hits were performed from 1 to 3m respectively.

The standardised drills were designed in consultation with experienced professional coaches outside the sampled team and the research team, as they were considered representative of commonly prescribed contact‐training practices across professional rugby league clubs and regularly used to reflect key elements of contact training. Drill selection, order, and setup were agreed upon prior to data collection and replicated exactly across all three sessions, with no modification to drill structure, constraints, or sequencing. All contact drills were completed in the same order on a grass surface. Visual examples of drill set‐up and order are provided in Figure 1.

*Wrestle drills* were performed from a starting position of either kneeling or standing (Figure 1a–b) were 1v1, and comprised of a takedown and grapple without submission. Players completed 3 × 30‐s rounds with maximal intent and had a minimum of 60 seconds rest between reps.

*Tackle shield hits* consisted of a 1v1 drill in which one player was the ‘ball‐carrier’ holding a shield while the other acted as the ‘tackler’. Ball‐carriers were asked to take a single step into contact without evasion, and tacklers were instructed to tackle with full intent (i.e., maximal effort given the constraints). Each rep initiated from the starting mark. Each player performed the tackler/carrier roles for 6 repetitions from 1 m and then 3 m apart respectively (Figure 1c).

*Ball‐carrier hits* were completed under the same conditions as those described during tackle shield hits with the ball‐carrier holding a ball instead of a shield (Figure 1d). Players were asked to carry the ball positioned on their chest.

The *ruck drill* was a 6v6 ‘open’ drill involving six tackles for both attacking and defending (Figure 1e). No kicking was allowed. Players started each repetition within a 20 × 10 m area, with the defensive line set 10 m from the play the ball. Players were instructed to initiate controlled contact with a cue for ‘bodies in front’. The ball‐carrier was taken to the ground before the next play the ball.

### 2.5 iMG Data Capture Framework

**FIGURE 2:**
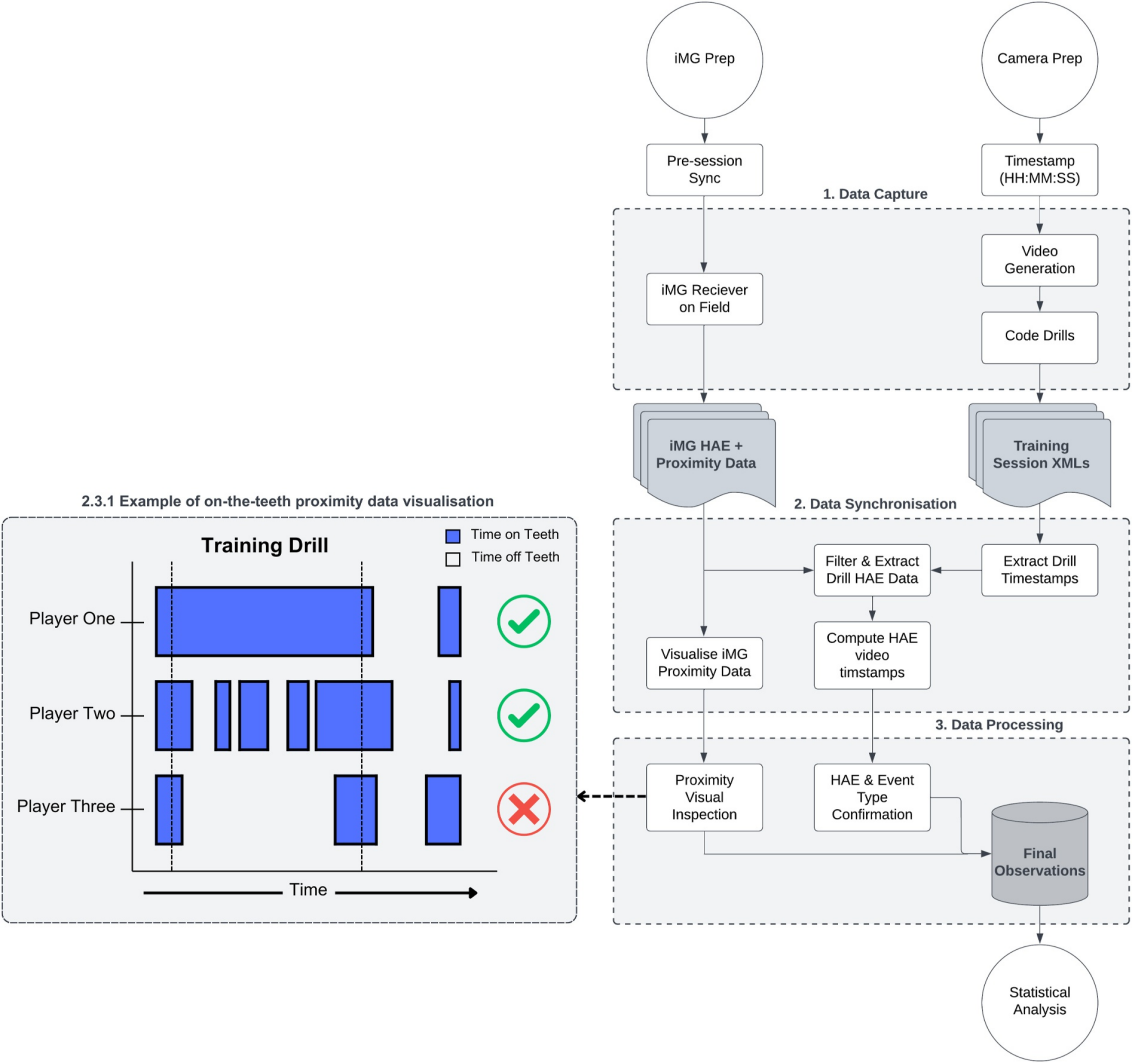
The iMG data capture framework with an example of on‐the‐teeth proximity data visualisation used during visual inspection. Example: Player one possessed complete on‐the‐teeth proximity data during the training drill period and thus met inclusion. Player two demonstrated intermittent use during the training drill period but had on‐the‐teeth proximity data for most of the period and thus met inclusion. A data check or later video verification would be used to ensure that any HAE recorded occurred during the player's time on teeth intervals. Player three would not meet the inclusion criteria because they lacked adequate ‘time on teeth’ during the training drill period.

Figure 2 provides an overview of each stage of the iMG and video‐based data capture (Figure 2.1), synchronisation (Figure 2.2), and processing (Figure 2.3) framework.

#### 2.5.1 Data Capture

Before the session, iMGs were fully charged and synchronised to the Prevent Biometrics mobile application. iMG data were transferred via Bluetooth continuously throughout the session and immediately uploaded to the cloud post‐session. A single video camera was used in an overhead position overlooking the training area (approximately 3 hours of video data were collected during the study). The camera zoom was adjusted between drills so that the demarcated training area for each drill was centred (i.e., all participants were continuously in frame). Upon recording initiation, a timestamp app (World Clock) was held to the camera to ensure the camera's first recorded frame represented the session's sync point (HH: MM: SS). This enabled accurate synchronisation of the iMG and video data. Post‐session, the primary researcher coded rep‐level drill‐event involvements for each player (i.e., each tackle, carry, or wrestle engagement) during the standardised drills using video analysis software (Hudl Sportscode), generating an XML file for each training session. Each discrete repetition/engagement (tackle, carry, or wrestle) was treated as a single coded drill‐event observation, meaning multiple observations could be recorded per player within each drill and session. For the subsequent data synchronisation stage, HAE data and on‐the‐teeth proximity sensor data (i.e., time‐on‐teeth) were exported from the Prevent Biometrics web portal in CSV format.

#### 2.5.2 Data Synchronisation

The XML files contained the start and end times of each drill relative to video time (i.e., elapsed video time, not real‐world time HH:MM:SS). A custom‐built R function (R build 402) converted video times to real‐world timestamps using the video sync point. These real‐world timestamps were used to filter and extract the training drill HAE data from the HAE data exports. This ensured that only HAE data associated with the standardised drills were included in subsequent analysis. Once extracted, the associated HAE timestamps were back‐transformed to video‐relative timestamps to allow later video verification. Finally, the proximity sensor data were visualised to depict each player's time‐on‐teeth data across each training session (Figure 2).

#### 2.5.3 Data Processing

To ensure that players wore an iMG during each drill, proximity sensor data were visualised and inspected (e.g., Figure 2.3.1). Three authors visually inspected the proximity sensor traces to determine whether players wore their iMG for each drill, and players were excluded from a drill if they lacked adequate proximity sensor data (see Figure 2.3.1 for an example). This ensured that only drill exposures (i.e., player present in a drill) and coded drill‐event observations (i.e., rep‐level tackle/carry/wrestle involvements) occurring during confirmed iMG wear (‘on‐teeth’) periods were included in the analysis, reducing the likelihood of underestimating HAE counts due to non‐wear periods. Although visual inspection is subjective, it was required to account for individual variation in iMG wearing behaviour (e.g., continuously worn, intermittently worn between repetitions, or removed during portions of a drill), which cannot be reliably captured using automated rules alone. Similar proximity‐sensor‐based approaches have previously been used to characterise iMG wear‐time and ‘on‐teeth’ periods in applied training environments (Roe, Sawczuk, Tooby, et al. 2024). Finally, HAE timestamps were synchronised with drill‐level video to confirm that each HAE occurred as a direct result of a drill event and could be attributed to a tackle, carry, or wrestle. To be included, an HAE was required to occur within 0.5 s of an identified drill event. The combined on‐teeth verification and drill‐event video attribution procedures ensured data integrity.

### 2.6 Statistical Analysis

Only drill‐linked HAEs recorded during confirmed on‐teeth periods were analysed. Overall drill‐level and drill event‐level HAE probabilities were estimated using binomial logistic regression models (R Core Team 2024; *stats* package). Drill‐level probability represented the likelihood of a player experiencing a HAE (≥ 5 *g*) during a drill exposure (i.e., while the player was participating in that drill). Drill event‐level probability represented the likelihood of a HAE (≥ 5 *g*) occurring within ± 0.5 s of a specific coded rep‐level drill event (i.e., tackle, carry, or wrestle). Probabilities (95% CI) were reported and expressed as percentages. Ordinal mixed‐effects regression models were used to calculate exceedance probabilities, defined as the probability that an HAE magnitude met or exceeded a given threshold during a drill event (Roe, Sawczuk, Owen, et al. 2024). Models included player and session dates as random effects. These probabilities estimate the likelihood of player involvement in HAEs meeting or exceeding a given magnitude threshold. Probabilities (95% CI) for PLA ≥ 25 *g* were reported as percentages. The 25 *g* threshold was chosen as an arbitrary cut‐off, as it is the lowest magnitude unlikely to be affected by linear acceleration trigger bias (Wang et al. 2021; Tooby, Till, et al. 2024a; Tooby, Woodward, Tucker, et al. 2024b). Use of this threshold also facilitates inter‐study comparison by aligning magnitude reporting with prior iMG literature. All statistical analyses were coded in R (version 4.3.0) using the *ordinal* (Christensen 2022) and *emmeans* (Lenth 2023) R packages.

## 3 Results

**FIGURE 3:**
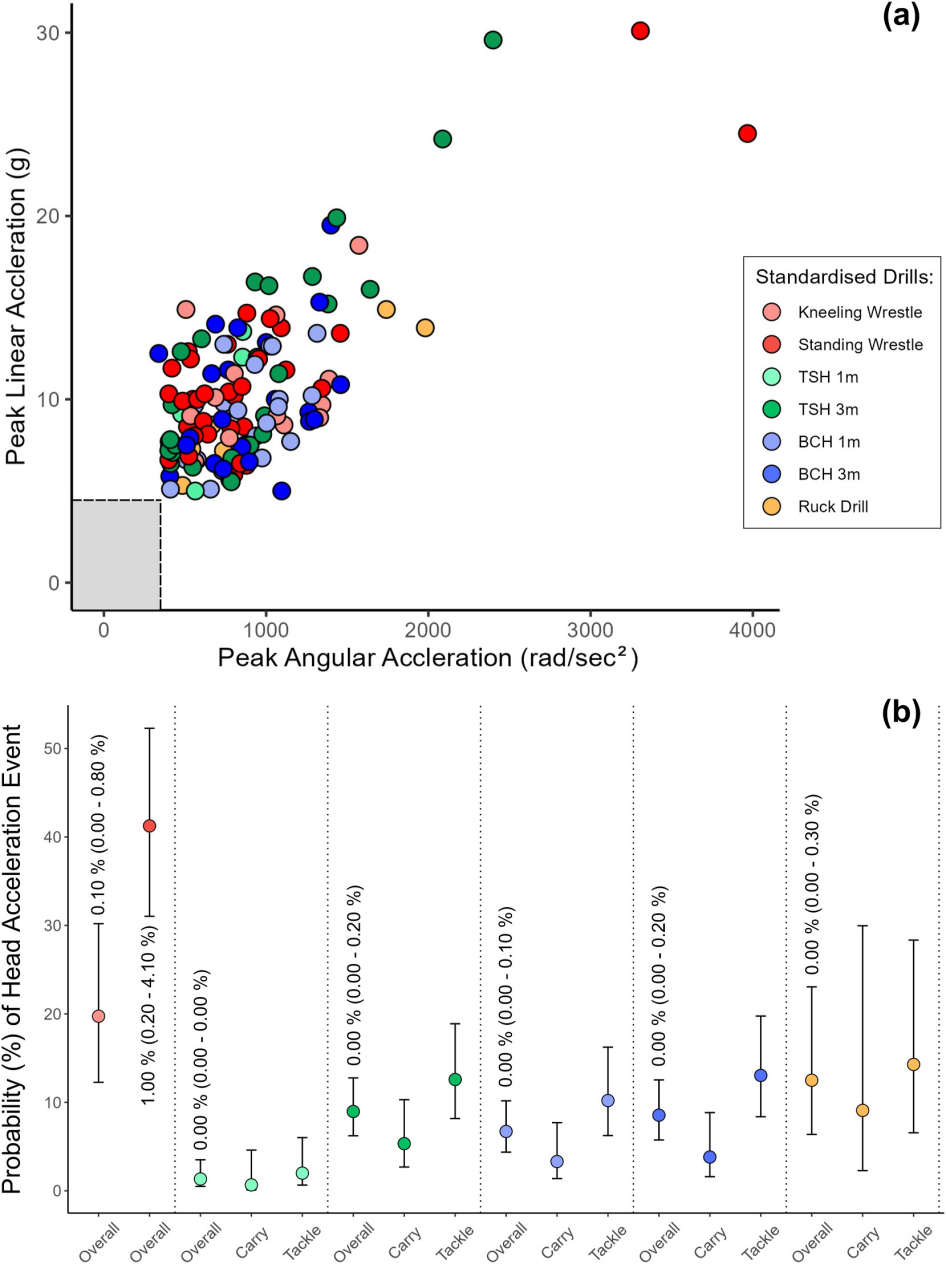
(a) Peak linear acceleration versus peak angular acceleration during common training drills (grey zone demonstrates trigger threshold) (≥ 5 *g* and ≥ 400 rad/s^2^), and (b) probability (95% CI) of HAE occurrence overall and per drill event. Overall drill ≥ 25 *g* exceedance probability (95% CI) indicated within each drill column. BCH = ball carrier hit; HAE = head acceleration event; TSH = tackle shield hit.

The number of drill event observations was 1402 (93 ± 50 per player), which resulted in 133 observed HAEs (≥ 5 *g*) (9 ± 8 per player). Fourteen drill observations and three HAEs were removed during data processing (Figure 2) due to insufficient on‐the‐teeth proximity data and non‐drill‐related HAE (e.g., slipping and falling during a retreat). Therefore, 1388 drill event observations and 130 HAEs were analysed further (wrestle = 48, tackler = 59, ball‐carrier = 23) (Figure 3a). The occurrence of multiple HAEs within a single drill event was rare, with only six drill events resulting in more than one HAE (kneeling wrestle = 2; standing wrestle = 4). The probability of a player experiencing a HAE (≥ 5 *g*) during each of the standardised drills and their component drill events (i.e., tackler vs. ball‐carrier) is depicted in Figure 3b. Standing wrestle had the highest probability of HAE (≥ 5 *g*) occurrence of 41.3% (CI = 31.0–52.3%) than other drills (range: 0.67%–14.3%). HAE probability was greater for tacklers than ball‐carriers. All drills were observed to have an exceedance probability of experiencing a HAE ≥ 25 *g*–0.0% (CI = 0.0–0.8%), except for standing wrestle 1.0% (CI = 0.2–4.1%) (Table 1 and Figure 3b).

**TABLE 1:** The overall probability (95% CI) of experiencing a HAE (≥ 5 *g*) and the probability of exceeding a given peak linear acceleration (*g*) threshold during common training drills.

Drill	Count: Drill events	Count: HAEs	Overall probability (95% CI) of HAE ≥ 5 *g*	PLA threshold (*g*)	Count: HAEs	Exceedance Probability (95% CI)
Kneeling wrestle	76	15	19.7% (12.3–30.2)	*Recorded	15	15.5% (7.4–26.3)
				≥ 10	7	6.9% (2.8–13.6)
				≥ 25	0	0.1% (0.0–0.8)
Standing wrestle	80	33	41.3% (31.0–52.3)	*Recorded	33	37.9% (25.6–51.2)
				≥ 10	20	21.7% (13.2–33.3)
				≥ 25	1	1.0% (0.2–4.1)
TSH 1m	300	4	1.3% (0.5–3.4)	*Recorded	4	0.9% (0.2–2.8)
				≥ 10	2	0.2% (0.0–0.9)
				≥ 25	0	0.0% (0.0–0.0)
TSH 3m	301	27	9.0% (6.2–12.8)	*Recorded	27	7.3% (3.9–12.5)
				≥ 10	12	2.7% (1.2–5.5)
				≥ 25	1	0.0% (0.0–0.2)
BCH 1m	298	20	6.7% (4.4–10.2)	*Recorded	20	4.9% (2.4–8.8)
				≥ 10	7	1.7% (0.7–3.5)
				≥ 25	0	0.0% (0.0–0.1)
BCH 3m	269	23	8.6% (5.7–12.5)	*Recorded	23	6.8% (3.6–12)
				≥ 10	10	2.5% (1.0–5.0)
				≥ 25	0	0.0% (0.0–0.2)
Standing wrestle	64	8	12.5% (6.4–23.1)	*Recorded	8	7.5% (2.9–17.3)
				≥ 10	2	2.8% (0.9–8.3)
				≥ 25	0	0.0% (0.0–0.3)

*Note:* Recorded = HAE met trigger threshold of ≥ 5 *g* and ≥ 400 rad/s^2^.

Abbreviations: BCH = ball carrier hit; PLA = peak linear acceleration; TSH = tackle shield hit.

## 4 Discussion

This study quantified the probability of HAEs experienced by professional rugby league academy players during standardised contact training drills. Across all drills and player roles, the probability of recording a HAE (≥ 5 *g*) during an individual drill event was generally low; however, meaningful variability was observed between drill types and player roles. Standing wrestle demonstrated the highest probability of HAE occurrence (41.3%, CI = 31.0–52.3%), whereas all other drills showed substantially lower probabilities (range: 0.67%–14.3%). Role‐specific differences were also evident, with tacklers demonstrating a greater probability of sustaining a HAE than ball‐carriers across most drill conditions. Despite these differences in overall HAE probability, the likelihood of higher‐magnitude events (≥ 25 *g*) was very low across drills (approximately 0.0%, CI = 0.0–0.8%), with standing wrestle again representing the highest exceedance probability (1.0%, CI = 0.2–4.1%). Collectively, these findings indicate that while most controlled contact events in training do not result in measurable head acceleration, specific drill types, particularly those involving sustained grappling contact, are associated with greater HAE probability. For context, match‐play analysis in professional rugby league have reported that the probability of recording a HAE during a tackle event is substantially higher, with approximately 40.3% of ball‐carrier and 33.2% of tackler involvements exceeding 10 *g*, and 6.3% and 4.3% exceeding 25 *g*, respectively. These match‐play values provide an important reference point when interpreting the present findings, suggesting that although certain training drills (e.g., standing wrestle) may elevate HAE probability relative to other training tasks, the majority of controlled training contacts occur within a lower overall exposure context than competitive match collisions (Tooby et al. 2025). These results provide initial evidence suggesting that constraint‐based drill design may influence the distribution of HAE exposures within training environments, while also demonstrating the utility of a synchronised, video‐verified iMG data‐capture framework for generating reliable event‐level exposure estimates.

Role‐specific differences in HAE probability were observed, with tacklers demonstrating a consistently higher likelihood of experiencing a HAE than ball‐carriers across most standardised drill conditions. This finding contrasts with some match‐play observations, where ball‐carriers have been reported to experience greater HAE involvement, and likely reflects the contrasting task constraints between competitive and controlled training environments (Tooby et al. 2025). Within the present standardised drills, ball‐carriers were frequently stationary or operating from controlled starting positions, whereas tacklers typically generated the majority of the approach momentum and collision force. This asymmetry in movement demands may increase the likelihood of measurable head acceleration for the tackler during the initial contact phase. In addition to differences in approach velocity, technical coaching objectives within controlled drills, such as emphasising dominant contact initiation or controlled body positioning, may further shape the mechanical characteristics of collisions and contribute to the observed role‐specific exposure patterns. These findings highlight the importance of considering task design, movement context, and role‐specific demands when interpreting HAE exposure data, as contact exposure characteristics observed in structured training settings may differ substantially from those observed during match‐play.

Beyond role‐specific differences, substantial variation in HAE probability was observed between drill types, with standing wrestle demonstrating markedly higher probabilities than all other standardised contact activities. The elevated HAE likelihood observed in wrestling drills is likely attributable to the unique mechanical and temporal characteristics of grappling‐based contact. Unlike discrete collision events that occur over short time intervals and allow momentum dissipation following impact, wrestling involves prolonged upper‐body engagement, repeated short‐duration contacts, constrained head positioning, and frequent attempts to gain leverage or dominant body position. These characteristics increase the opportunity for multiple head accelerations to occur within a single drill exposure, thereby elevating the probability of recording at least one HAE event. In contrast, tackle, carry, and open controlled contact drills typically involve clearly defined collision phases separated by recovery periods, limiting the number of contact repetitions per event and reducing the likelihood of repeated head acceleration exposure. Consistent with this interpretation, the probability of higher‐magnitude HAEs (≥ 25 *g*) remained extremely low across all drills, including wrestling, indicating that the observed differences between drills were primarily driven by increased occurrence of lower‐magnitude contact‐related head accelerations rather than an increased frequency of high‐magnitude impacts.

Additional drill‐level differences were observed between shielded and unshielded contact conditions. Tackle shield contacts performed from a 1 m approach distance demonstrated slightly lower HAE probabilities than equivalent unshielded contacts, suggesting that the padding may attenuate some impact transmission under low‐momentum collision conditions. However, this difference was not evident at the 3 m approach distance, where increased approach velocity and collision momentum may reduce the relative protective influence of the padding and minimise any observable attenuation effect. Although the absolute differences between conditions were modest and overall HAE probability remained low, these findings highlight how relatively small modifications to drill configuration, such as approach distance or the inclusion of protective equipment, can subtly alter the mechanical characteristics of contact exposure.

From an applied perspective, the observed variability between drills indicates that training‐task design and constraint manipulation may influence the distribution of HAE exposure within training sessions. Importantly, the present findings do not suggest that the majority of standardised contact drills are high‐risk, as the absolute probability of HAE occurrence remained low across most conditions. Rather, the results demonstrate that specific drill types, particularly those characterised by sustained grappling exposure, may contribute disproportionately to contact‐related head acceleration patterns. In applied sport science practice, drill classification libraries are frequently used to help practitioners select or sequence training activities according to targeted physical or mechanical exposure characteristics (e.g., locomotor intensity, acceleration load, or contact density). Extending this approach to include HAE probability profiling may enable practitioners to better understand how different training activities contribute to overall exposure patterns and, where appropriate, avoid unnecessary repetition of drills associated with relatively higher contact density while maintaining required technical and tactical training outcomes. While restricting individual drills alone would likely produce only modest reductions in total HAE accumulation, integrating drill‐level exposure information within a broader audit of the training process may support more informed training design decisions.

The contribution of individual drills to total HAE accumulation is likely determined not only by the probability of HAE occurrence per event but also by the relative volume of time allocated to each activity within a training microcycle. For example, although wrestling drills demonstrated substantially higher HAE probabilities, these activities typically occupy a smaller proportion of total weekly training exposure, meaning their overall contribution to cumulative HAE counts may be comparable to lower‐probability drills that are performed more frequently. Consequently, event‐level probability estimates should be interpreted alongside drill frequency, duration, and sequencing across the training week when evaluating potential exposure‐management strategies. Future research combining drill‐specific probability estimates with detailed time‐motion exposure data may enable more precise modelling of weekly or seasonal HAE accumulation within elite rugby league training environments.

Methodologically, the event‐linked iMG processing framework applied in this study provides additional support for previously established video‐synchronised and proximity‐verified approaches to HAE identification (Tooby, Woodward, Tucker, et al. 2024b). By ensuring accurate synchronisation with video footage, confirmation of on‐the‐teeth sensor proximity, and drill‐event linkage, the framework strengthens confidence in the validity of the HAE dataset analysed and enables consistent estimation of drill‐ and event‐specific HAE probabilities. Rather than replacing existing detection procedures, this structured verification approach contributes procedural transparency and reinforces the methodological robustness of event‐based HAE quantification within training research. Such standardised processing workflows may facilitate comparison across future studies and support continued refinement of methodological standards in contact‐exposure monitoring research.

## 5 Limitations

This study has several limitations that should be acknowledged when interpreting the findings. First, the sample was small and recruited via convenience sampling, primarily due to logistical and practical challenges encountered during recruitment. As such, the results should not be considered generalisable to the wider rugby league population. Future studies with larger, more diverse cohorts are necessary to enhance external validity. Second, as with all iMG systems that rely on linear acceleration‐based trigger mechanisms, there is a known risk of trigger bias, which may lead to false negatives. Specifically, certain impact conditions, particularly those involving contact to the front or crown of the head, can result in underestimation of linear acceleration at the sensor location, relative to the head's CoG. This technical limitation, documented in previous studies, may lead to under‐reporting of some HAEs, particularly those with lower than threshold PLA but still relatively high PAA (Wang et al. 2021; Tooby, Till, et al. 2024a). This interpretation aligns with field‐based validation findings reporting a sensitivity of 0.75 for the Prevent Biometrics iMG, suggesting that some true HAEs may not be recognised by the device under certain conditions (Jones et al. 2022). Consequently, the results of this study are likely an underestimation of the true in vivo HAEs. Third, the study population consisted solely of professional male academy players. While this group represents an important developmental stage within elite rugby league systems, further work is needed to explore HAE training exposure across broader populations, including senior professionals, females, and athletes in other sports or contact environments. Expanding the demographic reach of this methodology is essential for understanding how sex, playing level, and age influence HAE probability and incidence during training. Finally, due to the limited sample size, the analysis was conducted at the drill‐event level rather than at the individual player level. While this approach provides valuable insights into drill‐specific HAE probabilities, previous research has highlighted substantial inter‐individual variability in HAE probability and incidence (Tooby et al. 2025). Future work should seek to capture and analyse individual‐level differences to better inform individualised approaches to HAE and contact load management.

## 6 Conclusion

This study provides novel drill‐specific estimates of HAE probability during standardised contact training drills in professional rugby league academy players. Across all drills, the probability of recording a HAE during an individual contact event was generally low, and the likelihood of higher‐magnitude events (≥ 25 g) was extremely low under the standardised training conditions examined. These findings indicate that most contact exposures in these training drills are unlikely to result in measurable HAEs. Despite the overall low probabilities observed, variability was evident between drill types. Standing wrestle drills showed the highest probability of HAE occurrence, suggesting that activities characterised by prolonged grappling and repeated short‐interval contacts may contribute disproportionately to contact‐related head acceleration exposure compared with other contact drills, which showed similarly low probabilities across conditions. Role‐specific differences were also evident, with tacklers generally experiencing higher HAE probabilities than ball‐carriers within the standardised drill formats. While the low baseline probability of HAE occurrence suggests that modifying individual drills alone is unlikely to produce substantial reductions in overall HAE accumulation, profiling drill‐specific exposure characteristics may help practitioners better understand how different training activities contribute to the distribution of contact exposures within the training process.

## Funding

This project was funded by Prevent Biometrics and the Rugby Football League as part of Leeds Beckett University's TaCKLE (Tackle and Contact Kinematics, Load and Exposure) project.

## Disclosure

R.W. PhD was part‐funded by Prevent Biometrics, C.O. and J.T. research fellowships are part‐funded by the RFL, GR research fellowship is part‐funded by PREM Rugby, G.P. and P.A. are employed by the RFL, D.V. is employed in a consultancy capacity by the RFL, B.J. is employed in a consultancy capacity by the RFL and PREM Rugby.

## Conflicts of Interest

The authors declare no conflicts of interest.

## Data Availability

Research data are not shared.
